# Eugenol: A Phyto-Compound Effective against Methicillin-Resistant and Methicillin-Sensitive *Staphylococcus aureus* Clinical Strain Biofilms

**DOI:** 10.1371/journal.pone.0119564

**Published:** 2015-03-17

**Authors:** Mukesh Kumar Yadav, Sung-Won Chae, Gi Jung Im, Jae-Woo Chung, Jae-Jun Song

**Affiliations:** 1 Institute for Medical Device Clinical Trials, Korea University College of Medicine, Seoul, South Korea; 2 Department of Otolaryngology—Head and Neck Surgery, Korea University College of Medicine, Seoul, South Korea; 3 Laboratory of Medicine, Dongguk University Ilsan Hospital, Goyang, Gyeonggi, South Korea; Institut Pasteur, FRANCE

## Abstract

**Background:**

Inhibition and eradication of *Staphylococcus aureus* biofilms with conventional antibiotic is difficult, and the treatment is further complicated by the rise of antibiotic resistance among *staphylococci*. Consequently, there is a need for novel antimicrobials that can treat biofilm-related infections and decrease antibiotics burden. Natural compounds such as eugenol with anti-microbial properties are attractive agents that could reduce the use of conventional antibiotics. In this study we evaluated the effect of eugenol on MRSA and MSSA biofilms *in vitro* and bacterial colonization *in vivo*.

**Methods and Results:**

Effect of eugenol on *in vitro* biofilm and *in vivo* colonization were studied using microtiter plate assay and otitis media-rat model respectively. The architecture of *in vitro* biofilms and *in vivo* colonization of bacteria was viewed with SEM. Real-time RT-PCR was used to study gene expression. Check board method was used to study the synergistic effects of eugenol and carvacrol on established biofilms. Eugenol significantly inhibited biofilms growth of MRSA and MSSA *in vitro* in a concentration-dependent manner. Eugenol at MIC or 2×MIC effectively eradicated the pre-established biofilms of MRSA and MSSA clinical strains. *In vivo*, sub-MIC of eugenol significantly decreased 88% *S*. *aureus* colonization in rat middle ear. Eugenol was observed to damage the cell-membrane and cause a leakage of the cell contents. At sub-inhibitory concentration, it decreases the expression of biofilm-and enterotoxin-related genes. Eugenol showed a synergistic effect with carvacrol on the eradication of pre-established biofilms.

**Conclusion/Major Finding:**

This study demonstrated that eugenol exhibits notable activity against MRSA and MSSA clinical strains biofilms. Eugenol inhibited biofilm formation, disrupted the cell-to-cell connections, detached the existing biofilms, and killed the bacteria in biofilms of both MRSA and MSSA with equal effectiveness. Therefore, eugenol may be used to control or eradicate *S*. *aureus* biofilm-related infections.

## Introduction

*Staphylococcus aureus* is one of the major human pathogens responsible for the infections of skin, soft tissue, respiratory tissue and bone joints, causing endovascular infections such as bacteremia, endocarditis, sepsis, and toxic shock syndrome [1]. It is one of the most common pathogens implicated in a wide variety of biofilm-related infections, including infections involving implanted medical devices such as bloodline catheters and heart implants, and infections associated with cystic fibrosis, wounds, superficial skin infections, and otitis media [2,3,4,5,6]. In the adhesive state, bacteria attach to the surface of biomaterials or tissues and form a multilayered structure consisting of slow-growing bacteria enclosed in an extracellular polymeric matrix [7]. The physiology of cells in biofilms is different from that of planktonic cells, and the expression levels of a number of genes are reported to be enhanced exclusively in biofilm formation. Staphylococcal biofilm accumulation is mediated by polysaccharide intercellular adhesion (PIA), modulated by gene products encoded by *ica* operon [8,9]. However, many researchers reported that *S*. *aureus* can form *ica*-independent biofilm, mediated by proteins binding to proteins of the extracellular matrix [10,11]. Similarly *sarA* gene expression has been reported to promote biofilm formation in *S*. *aureus* [12,13].

The bacteria in biofilms are slow-growing and were found to be particularly resistant to antibiotic treatment [14]. As a result conventional antibiotic therapy is ineffective in inhibition and eradication of biofilms infections. The treatment is further complicated by the rise of antibiotic resistance among *staphylococci*. Consequently, there is a need for novel antimicrobials that can treat biofilm-related infections, are safe to use, and limit the emergence of antibiotic resistant bacteria. One of the emerging concepts in infection treatment is the disruption of the bacterial membrane bilayer or the proteins integral to membrane function in dormant bacteria of biofilms [15].

Only few antimicrobials are known to be capable of disrupting the cell membrane and are effective against *S*. *aureus* biofilm-related infection. Daptomycin and telavancin are the two antibiotics clinically approved in 2003 and 2009, respectively, for use against *S*. *aureus* biofilm infection [16]. However, it was reported that daptomycin may not be able to eradicate all types of *staphylococcal* biofilm infections in a mouse model [17]. Various phyto-compounds such as 220D-F2 derived from *Rubus ulmifolius* plant, carvacrol (constituent of oregano), tea tree oil (*Melaleuca alternifolia*), and magnolol have been reported to be effective against *S*. *aureus* biofilms [18,19,20,21,22].

Eugenol (4-allyl-2-methoxyphenol) is a major component of clove oil and has been used as a flavoring agent in food and cosmetic products. Studies have shown that eugenol exhibits a number of potentially beneficial biological properties, including antimicrobial, antioxidant, anti-inflammatory, anticarminative, and antispasmodic activities [23,24,25,26]. Recently, we demonstrated biofilm inhibition and eradication properties of eugenol against *streptococci* [27]. Previous studies have also demonstrated that eugenol can disrupt the cell membrane of *Salmonella typhi*, and decreases toxin-related gene expression of *S*. *aureus* at sub-inhibitory concentrations [28,29]. However, the effect of eugenol on methicillin-resistant and methicillin-sensitive *S*. *aureus* (MSSA and MRSA respectively) clinical strain biofilms has not been studied. In this study, we aimed to evaluate the effect of eugenol on MRSA and MSSA biofilms *in vitro* and on bacterial colonization using *in vivo* otitis media (OM) model.

We demonstrated here that eugenol exhibits notable activity against MRSA and MSSA clinical strain biofilms. Eugenol inhibited biofilm formation, disrupted the cell-to-cell connections, detached the existing biofilms, and killed the bacteria in biofilms of both MRSA and MSSA with equal effectiveness. It decreased the gene expression of biofilm and enterotoxin genes, and decreased bacterial colonization in the middle ear. Therefore, eugenol alone or in combination with carvacrol may be used to control or eradicate *S*. *aureus* biofilm-related infections.

## Materials and Methods

### Ethics statement

All animal experiments were carried out in accordance with the guidelines of the Animal Research Committee Dongguk University Ilsan Hospital. The animal experiments were approved by Animal Research Committee Dongguk University Ilsan Hospital, Gyeonggi, South Korea. The bacterial experimental protocol was approved by the Institutional Review Board Dongguk University Ilsan Hospital, Gyeonggi, South Korea (Permit Number: 2013-50). Bacterial strains were isolated from clinical samples collected from patients visiting Dongguk University Ilsan Hospital (June 2013-January 2014). And all patients provided written informed consent for the collection of samples and subsequent analysis.

### Bacterial strains and culture conditions

MSSA strain ATCC 29213 was obtained from the American Type Culture Collection (ATCC, USA). Seventy two *S*. *aureus* clinical strains (43 MRSA and 29 MSSA) were isolated and identified at the department of clinical microbiology, Dongguk University Ilsan Hospital, Goyang, South Korea by using standard procedures. These clinical strains were isolated from the following samples: blood (*n* = 10); sputum (*n* = 10); wound infections (*n* = 10); pus (*n* = 10); ear discharge (*n* = 10); nasal swab (*n* = 7); urine (*n* = 4); and other sites, such as catheters (*n* = 11). Five MRSA strains (CCARM 3903, CCARM 3108, CCARM 3912, CCARM 3967, and CCARM 3807) resistant to oxacillin (≥128.0 μg/mL), erythromycin (≥128.0 μg/mL), clindamycin (≥128.0 μg/mL), and gentamicin (32–128.0 μg/mL) were purchased from Culture Collection of Antimicrobial Resistant Microbes (CCARM), Seoul, Korea (S1 Table). Mueller-Hinton broth (MHB), tryptic soya broth (TSB), and BHI agar were purchased from BD Biosciences, Inc. (Sparks, MD, USA). Eugenol (oil) was purchased from Sigma-Aldrich (St. Louis, MO, USA), and stock solutions were prepared in dimethyl sulfoxide (DMSO) (Sigma-Aldrich).

### Determinations of minimum inhibitory concentration (MIC) and minimum bactericidal concentration (MBC)

MICs and MBCs were determined using the broth micro-dilution method as recommended by the Clinical and Laboratory Standards Institute [30]. Cultures of *S*. *aureus* in MHB prepared overnight at 37°C with shaking were diluted 10-fold in fresh MHB and incubated at 37°C with shaking until they reached the exponential-growth phase. Serial 2-fold dilutions of eugenol resulting in concentrations ranging from 0.01% to 0.04% were prepared in MHB in a 96-well plate (200 μL per well). Wells with no added eugenol were used as positive controls. All wells contained 0.1% v/v DMSO to increase the solubility of eugenol. A diluted bacterial suspension was added to each well to give a final concentration of 1–5×10^5^ colony-forming units (cfu)/mL, as confirmed by viable counts. In each MIC and MBC experiment wells without added bacteria (media only) were used as negative controls. The plates were incubated for 24 h at 37°C and growth was assessed by measuring the optical density at 600 nm (OD_600_) by using a spectrophotometer. Aliquots (10 μL) obtained from the wells showing no visible growths were plated on BHI agar and the number of colonies was counted following overnight incubation at 37°C. The MIC was defined as the lowest eugenol concentration with no visible growth, and the MBC was defined as the lowest concentration that was observed to reduce the initial inoculum by ≥99.9%. The experiments were performed in triplicates and repeated 3 times.

### Effect of eugenol on biofilm growth

The biofilm assay was performed using 24- or 96-well polystyrene flat-bottom microtiter plates (BD falcon, Sparks, MD, USA) in a static model, using a previously described procedure [31]. Overnight cultures of *S*. *aureus* were diluted 1:200 with fresh sterile TSB medium containing 0.5% glucose. For the screening of test compounds, 200-μl aliquots of the diluted cultures were transferred to the wells of 96-well microtiter plates with eugenol or DMSO controls performed in triplicate. Eugenol diluted by serial 2-fold dilutions (final concentration ranging from 0.01% to 0.08%) were added to the cell suspensions. Cell suspensions (200 μL) were inoculated in 96-well microtiter plate and incubated for 24 h at 37°C. After incubation, the medium was discarded, and plates were gently washed three times with sterile PBS. Thereafter, plates were stained with 50 μL of crystal violet (0.1%) for 15 min. Excess stain was removed by washing with PBS. The crystal violet (CV) attached to the biofilm samples were dissolved with 200 μL of ethanol. The absorbance at 570 nm (OD_570_) was measured using a spectrophotometer (Molecule Probe) as the value of biofilm formation. Inhibition of CV staining by the drug treatment was calculated as a percentage of the mean of DMSO-treated control samples. Experiments were repeated three times to obtain the mean and standard deviation of means of biofilm formation under each treatment.

### Effect of eugenol on established biofilms

The biofilm formation ability of all 72 clinical strains (43 MRSA and 29 MSSA) was evaluated by CV-microtitter plate assay as described above in separate experiments (S1 Fig.). High biofilm-forming clinical isolates (*n* = 26) were selected for further study. To test the effect of eugenol on established biofilms, biofilms that had been pre-formed for 24 h were washed with PBS to eliminate planktonic bacteria and exposed to indicated concentrations of eugenol for 6 h at 37°C. The biofilms were then washed again with PBS, dispersed by sonication, and dissolved in 100 μL PBS. The dispersed biofilm cells were used to determine viable cfu per mL from diluted samples plated and grown on BHI agar. The biomass of the biofilm was detected by the CV-microtiter plate method, as described above.

### Determination of rate of kill bacteria within biofilms

The rate of kill of *S*. *aureus* within biofilms upon treatment with eugenol was evaluated as described earlier [28]. *S*. *aureus* (ATCC 29213) biofilms pre-formed over 24 h were treated in 24-well plate with 0.04% (MIC) and 0.08% (2×MIC) of eugenol for 0, 15, 30, 60, 180, and 360 min. Control biofilms were prepared using identical treatments without eugenol. The biofilms were washed and cfu were counted. All the determinations were done in triplicates and repeated three times.

### Scanning electron microscopic analysis of biofilm

Scanning electron microscopy (SEM) was used to observe the architecture of *S*. *aureus* biofilms grown in the presence of eugenol (0.5 × MIC concentration) and to evaluate the eradication of established biofilms treated with eugenol. The cell suspension was prepared as described above and 1.5 mL cell suspensions were inoculated in a 24-well microtiter plate and incubated at 37°C for 24 h. For SEM analysis of biofilm eradication, the media of pre-established biofilms were replaced with fresh media containing eugenol (0.04%), and further incubated for 6 h. The plate was gently washed twice with sterile PBS to remove the planktonic cells and preserved in SEM solution. The samples were pre-fixed by immersion in 2% glutaraldehyde in 0.1 M phosphate buffer, and post-fixed for 2 h in 1% osmic acid dissolved in PBS. The samples were treated in a graded series of ethanol and t-butyl alcohol, dried in a freeze dryer (ES-2030, Hitachi, Tokyo, Japan), platinum-coated using an ion coater (IB-5, Eiko, Kanagawa, Japan), and observed under a Field Emission-Scanning Electron Microscope (FE-SEM; Hitachi, S-4700, Tokyo, Japan).

### *In vivo* colonization using OM rat model

Twenty seven pathogen-free Sprague-Dawley rats weighing 150–200 g were obtained from Orient Bio (Gyeonggi, South Korea). All animals were examined for middle ear abnormalities prior to the experiment and were kept isolated in an infection-free zone for 2 weeks. The rats were assigned randomly to groups that received bacteria only (*n* = 8), bacteria with 0.5×MIC eugenol (*n* = 8), media with DMSO (DMSO control; *n* = 7) or no procedure (*n* = 4). The bacterial cell suspension was prepared in TSB medium supplied with DMSO at the final concentration of 0.1% to dissolve eugenol. The cytotoxicity of DMSO on human middle ear epithelium cell (HMEEC) line and *in vivo* was evaluated separately by CCK-8 kit (Dojindo, Maryland, USA) and histological analysis. And no significant effect of 0.1% DMSO was detected on HMEEC line and *in vivo* (S2 & S3 Fig.). Cell suspension aliquots (50 μL) containing 3×10^7^cfu *S*. *aureus* (ATCC 29213) were injected into the middle ear cavity through the tympanic membrane of the right ear using a tuberculin syringe and a 27-gauge needle [32]. The animals were euthanized using a 1:1 combination of ZoletilH (tiletamine-zolazepam, Virbac, Carros, France) and RompunH (xylazine-hydrochloride, Bayer, Leverkusen, Germany) 1 week after inoculation, and the bulla was aseptically acquired. The tympanic membrane was removed and ears were irrigated to remove the planktonic bacteria. The bulla from each group were dissected and cleaned, and the visualized middle ear was photographed. The bulla were homogenized, serially diluted, and plated on blood agar plates (BAP) to obtained cfu counts. For SEM analysis the bulla were preserved in SEM solution and analyzed as described above.

### Synergistic effects of eugenol and carvacrol on eradication of biofilms

The synergistic effects of eugenol and carvacrol were assessed by the checkerboard test as previously described [33]. MRSA and MSSA biofilms established over 24 h were treated with serial 2-fold dilutions of eugenol, carvacrol, or a combination of the two compounds for 6 h. After the 6 h incubation at 37°C, cfu were counted. The fractional inhibitory concentration index (FICI) was calculated as the sum of the FICs calculated for each of the drugs, which in turn was defined as the MIC of each drug when used in combination, divided by the MIC of the drug when used alone. The interaction was defined as synergistic if the FIC index was less than or equal to 0.5, additive if the FIC index was greater than 0.5 and less than or equal 1.0, indifferent if the FIC index was greater than 1.0 and less than or equal to 2.0, and antagonistic if the FIC index was greater than 2.0. Here Minimum biofilm eradication concentration (MBEC) was defined as the minimum eugenol concentration that elicits a ≥90% decrease in the number of viable cells in the established biofilm. The term MIC is replaced by MBEC for the calculations of FICI and FICs.

### Crystal violet absorption

The alteration in membrane permeability was detected by the crystal violet assay [28]. Suspensions of the ATCC 29213 strain were prepared in LB broth. Cells were harvested by centrifugation at 4500×g for 5 min at 4°C. The cells were washed twice and resuspended in PBS (pH-7.4). Eugenol and oxacillin at MIC and 2×MIC (MIC values for eugenol and oxacillin were 0.04% and 3.2 μL/mL, respectively) were added to the cell suspension and incubated at 37°C for 30 min. Control samples were prepared similarly without treatment with eugenol. The cells were harvested at 9300×g for 5 min. After that the cells were resuspended in PBS containing 10 μL/mL of crystal violet. The cell suspension was then incubated for 10 min at 37°C. The suspension was then centrifuged at 13,400×g for 15 min and the OD_590_ of the supernatant was measured in spectrophotometer. The OD value of the crystal violet solution, which was originally used in the assay, was taken and it was considered as 100%. The percentage of crystal violet uptake of all the samples was calculated using the following formula:

$$\frac{\text{OD value of the sample}}{\text{OD value of the crystal violet solution}}\times 100$$

### Quantification of gene expressions using Real-time RT-PCR

*S*. *aureus* (ATCC 29213) biofilms were grown for 24, 36,or 48 h in 12-well plate as described above at 37°C either with eugenol (at 0.5 × MIC) or without eugenol. RNA was prepared as described by Qiu et al [29]. Briefly, medium was discarded, biofilms were washed three times with PBS and cells were harvested by centrifugation (5,000 × *g* for 5 min at 4°C). The cell pellet was dissolved in 100 μL (100μg/mL) of lysostaphin (Sigma-Aldrich) for 10 min at 37°C. Total bacterial RNA was isolated using Qiagen RNeasy kit (Qiagen, Hilden, Germany) in accordance with the manufacturer’s instructions. The contaminating DNA was removed using the optional on-column RNase-free DNase I step (Qiagen). RNA concentrations were determined by OD_260_ measurements and the quality of RNA was checked on an RNase-free 2% agarose gel. cDNA synthesis was carried out using the ImProm-II Reverse Transcriptase Kit (Promega, Madison, WI, USA) according to the manufacturer’s instructions. Briefly, tailing of RNA with a random hexamer primer was performed at 70°C for 5 min, annealing at 25°C for 5 min, extension at 37°C for 1 h and inactivation of samples at 70°C for 15 min. The primer pairs used in real-time RT-PCR are listed in Table 1. Real-time RT-PCR was carried out in a total volume of 20 μL, consisting of 10 μL 2X SYBR Green PCR Master Mix (Roche Applied Science, Indianapolis, IN, USA), 2.5 pmol of forward and reverse primers, and 2 μL of cDNA. PCR conditions included initial denaturation at 95°C for 10 min, followed by 45 cycles of denaturation (95°C for 15 sec), annealing (56°C for 15 sec), extension (72°C for 15 sec) followed by final extension (72°C for 5 min) and melting curve analysis from 60–95°C. Negative controls containing nuclease-free water instead of c-DNA were run concomitantly to confirm that the samples were free from contamination. To verify the absence of contaminating genomic DNA, each RT-PCR experiment included a no reverse transcriptase control. The relative gene expression was analyzed using the 2^−ΔΔCT^ method [34]. The reference gene was 16S r-RNA and the standard condition was biofilms grown without eugenol.

**Table 1:** List of primers used for gene expression study.

Gene name	Sequence	Base pair	Amplicon size (base pair)
*icaD*	F-CGTGTTGCTTTAAACATTGAAAAT	24	126
	R- TCTTCCTCTCTGCCATTTTTG	21	
*seA*	F- ATGGTGCTTATTATGGTTATC	21	163
	R- CGTTTCCAAAGGTACTGTATT	21	
*sarA*	F-TTTCTCTTTGTTTTCGCTGATG	22	124
	R- TTGCTTTGAGTTGTTATCAATGG	23	
*16S*	F- GCTGCCCTTTGTATTGTC	18	178
	R-AGATGTTGGGTTAAGTCCC	19	

### Statistical analysis

Data were calculated as the means of individual experiments performed in triplicate and compared with those of the control groups. Statistical analysis was performed using 2-tailed Student’s *t*-test and one-way analysis of variance (ANOVA). Statistical significance was set at a p-value of less than 0.05.

## Results

### MIC and MBC of eugenol

**Fig 1:**
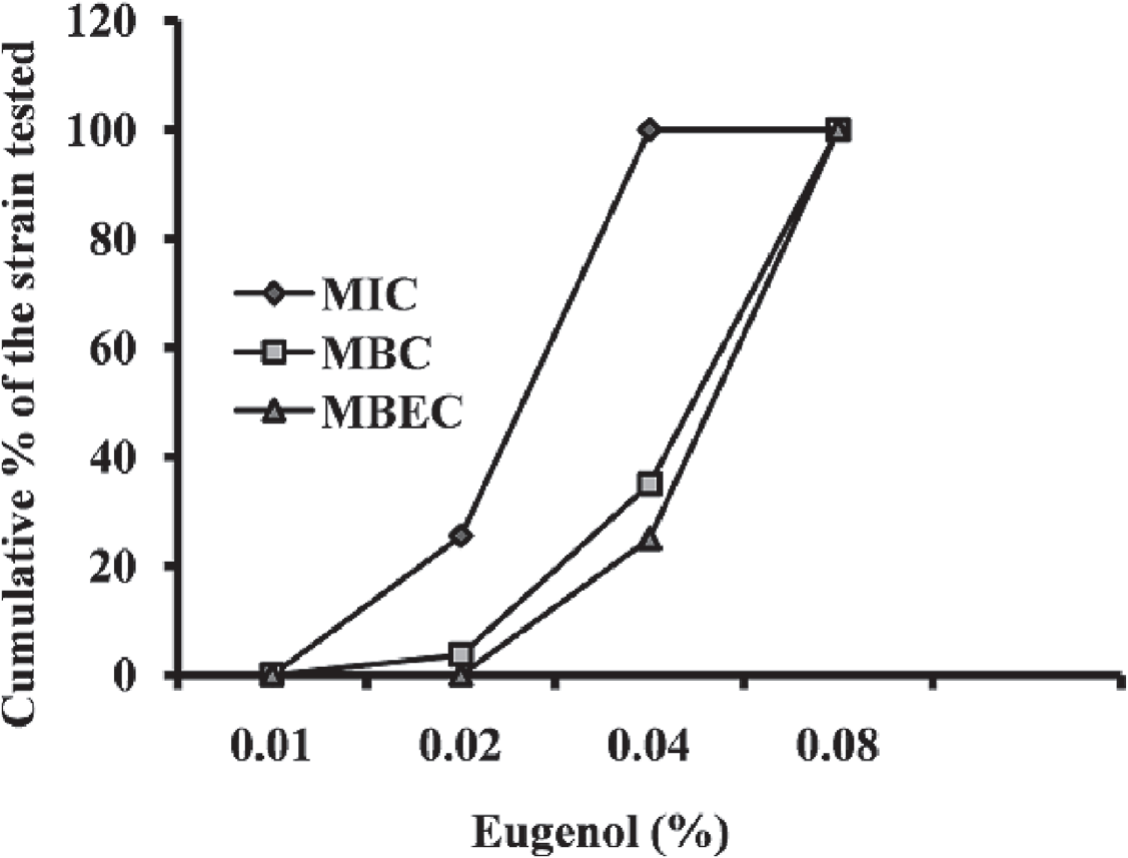
Cumulative minimum inhibitory concentration (MIC), minimum bactericidal concentration (MBC) and minimum biofilm eradication concentration (MBEC) of eugenol against *Staphylococcus aureus* expressed as a percentage of tested strains. MIC and MBC, *n* = 78; MBEC, *n* = 32.

The MIC values of eugenol for *S*. *aureus* ranged from 0.01% to 0.04%, with the MBC measured to be approximately double the MIC. No difference was observed in MIC values between the MRSA and MSSA isolates. The MIC, MBC, and MBEC of eugenol are presented in Fig. 1. No significant growth inhibitory activity of 0.1% DMSO was detected on *S*. *aureus* (S2 Fig.).

### Eugenol inhibits biofilm growth

**Fig 2:**
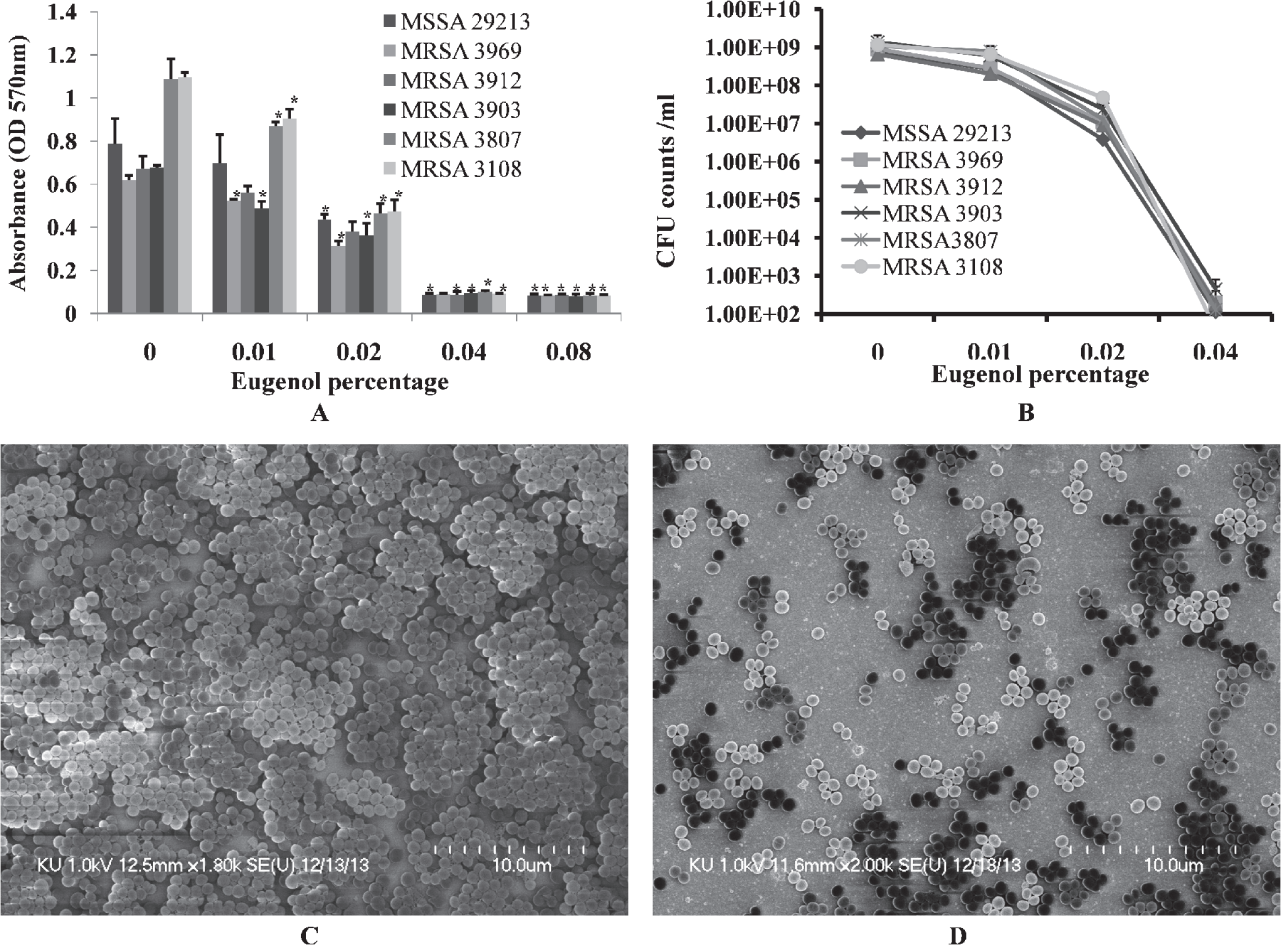
Inhibition of MRSA and MSSA biofilm growth *in vitro* with different concentrations of eugenol. (A) Decreased biofilm biomass detected using the microtiter plate assay. (B) Decreased cell viability within biofilms, detected by cfu counts. The error bars represent standard deviation from the mean value. (C) Representative SEM image of *S*. *aureus* (ATCC 29213) biofilm grown without eugenol supplement. (D) Representative SEM image of biofilms grown with 0.5×MIC eugenol. The SEM image scale bar corresponds to 10μm.

Eugenol significantly decreased MRSA and MSSA biofilm growth *in vitro*. At low concentrations, the decrease in biofilm growth was dose-dependent, reaching complete inhibition of biofilm formation at higher concentrations. Eugenol at concentration of 0.5×MIC decreased the biomass of MRSA (CCARM) and MSSA (ATCC 29213) isolates biofilms by more than 50% (Fig. 2A). The cfu of eugenol-supplemented biofilms were found to be significantly decreased approximately two log10 steps (Fig. 2B).

The architecture of *S*. *aureus* (ATCC 29213) biofilms grown with and without eugenol (at 0.5×MIC) were analyzed by SEM. The SEM analysis revealed significantly decreased biofilm growth in eugenol-supplemented sample in comparison to the untreated samples (Fig. 2C&D). The biofilms grown without eugenol were thick, organized in 3-dimensional structure, with the cells connected to each other. Conversely, the biofilms grown in the presence of eugenol were not well organized, with the cells scattered and remaining attached to the bottom of the microtiter plate. The results of the microtiter plate assay and cfu count indicates that eugenol kills or inhibits the bacterial growth consequently with low cells decreased biofilms were formed. As a result of low biofilms, the cell aggregation and cell-to-cell connections were prevented (as shown by SEM images), resulting in loosely arranged cell that can be easy disrupted.

### Eugenol eradicates established biofilms

**Fig 3:**
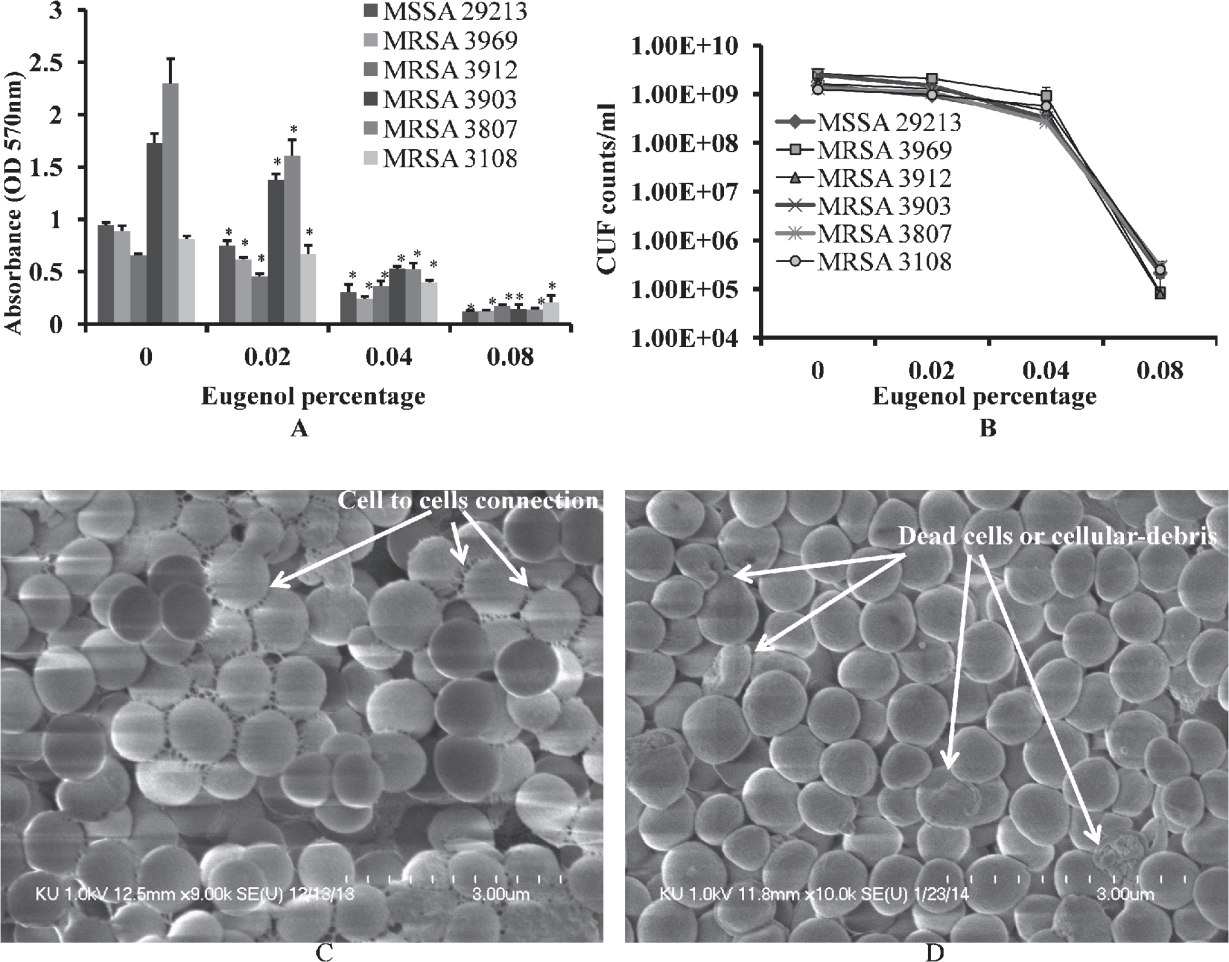
Eradication of established biofilms of MRSA and MSSA by a range of concentrations of eugenol. (A) Decreased biomasses of established biofilms, detected by microtiter plate assay. (B) Decreased cell viability of biofilm, detected by cfu counts. The error bars represent standard deviation from the mean value. (C) Representative SEM image of established untreated biofilm of *S*. *aureus* (29213). (D) Representative SEM image of established biofilm treated with eugenol (MIC). SEM images scale bar corresponds to 3μm.

Eugenol demonstrated strong anti-biofilm properties against both MRSA and MSSA clinical strains (Table 2). The biomasses of already established biofilms were significantly decreased upon eugenol treatment (Fig. 3A). Similarly, the number of viable bacteria were significantly decreased in eugenol-treated biofilms (Fig. 3B). Eugenol at MIC significantly decreased the biomass of already established biofilms by more than 50%. A decrease of four log 10 steps in the number of viable cells was observed in biofilm treated with 2×MIC eugenol. The MBEC of eugenol against biofilms from MRSA and MSSA clinical strains was found to be twice the MIC value.

**Table 2:** Effect of eugenol on the eradication of established biofilms of MRSA and MSSA clinical strains.

*S. aureus* strains	Mean OD_570_ (CV) of biofilms detected by microtiter plate assay							
	0% (Control)	SD	0.02%	SD^c^	0.04%	SD	0.08%	SD
**MSSA ATCC** ^b^ **29213**	0.9430	± 0.02	0.7485	±0.04	0.3072	±0.07	0.1233	±0.007
**MRSA CCARM** ^**a**^ **3969**	0.8896	± 0.05	0.6172	±0.01	0.2412	±0.02	0.1270	±0.007
**MRSA CCARM 3912**	0.6565	± 0.01	0.4565	±0.02	0.3602	±0.05	0.1710	±0.016
**MRSA CCARM 3903**	1.7317	± 0.08	1.3803	±0.05	0.5279	±0.02	0.1452	±0.042
**MRSA CCARM 3807**	2.2991	± 0.13	1.6092	±0.12	0.5256	±0.05	0.1435	±0.009
**MRSA CCARM 3108**	0.8122	± 0.02	0.6678	±0.08	0.3991	±0.01	0.1098	±0.035
**MRSA (Wound)**	0.9122	± 0.04	0.7942	±0.07	0.5913	±0.09	0.1468	±0.002
**MRSA (Nasal cavity)**	1.356	± 0.18	0.9549	±0.04	0.5150	±0.06	0.1443	±0.001
**MRSA (Nasal cavity)**	1.266	± 0.12	0.9566	±0.05	0.5177	±0.16	0.1479	±0.003
**MRSA (Wound)**	0.9443	± 0.08	0.5191	±0.03	0.4883	±0.03	0.1271	±0.016
**MRSA (catheter)**	0.9075	± 0.04	0.6906	±0.05	0.4997	±0.04	0.1087	±0.015
**MRSA (Wound)**	0.9603	± 0.05	0.7073	±0.06	0.5251	±0.02	0.1107	±0.012
**MRSA (Wound)**	1.0737	± 0.07	0.6989	±0.04	0.5251	±0.02	0.1131	±0.011
**MSSA (Blood)**	1.523	± 0.14	0.8527	±0.07	0.6448	±0.06	0.1547	±0.002
**MSSA (Blood)**	0.8546	± 0.09	0.5329	±0.07	0.4521	±0.01	0.1236	±0.023
**MRSA (Wound)**	0.9657	± 0.16	0.7506	±0.07	0.5402	±0.09	0.1505	±0.008
**MSSA (Pus)**	1.2114	± 0.15	0.8946	±0.05	0.6665	±0.01	0.1273	±0.004
**MSSA (Pus)**	0.8612	± 0.11	0.5628	±0.03	0.3428	±0.08	0.1262	±0.003
**MRSA (Wound)**	0.9651	± 0.12	0.5884	±0.06	0.4552	±0.07	0.1383	±0.007
**MRSA (Wound)**	0.9121	± 0.06	0.5384	±0.03	0.4218	±0.08	0.1324	±0.006
**MSSA (Ear discharge)**	0.8621	± 0.05	0.6747	±0.02	0.4678	±0.05	0.1274	±0.012
**MRSA (Blood)**	0.9062	± 0.05	0.6751	±0.07	0.4553	±0.06	0.1648	±0.034
**MRSA (Ear discharge)**	1.1972	± 0.19	0.7403	±0.006	0.4654	±0.06	0.1467	±0.035
**MSSA (Ear discharge)**	0.9331	± 0.07	0.7581	±0.09	0.5686	±0.11	0.1254	±0.010
**MRSA (Urine)**	1.2712	± 0.25	0.7011	±0.03	0.4452	±0.05	0.1143	±0.003
**MSSA (Ear discharge)**	1.1241	± 0.11	0.8771	±0.02	0.7200	±0.05	0.1379	±0.014
**MSSA (blood)**	1.0931	± 0.11	0.6723	±0.05	0.4743	±0.16	0.1464	±0.034
**MSSA (Blood)**	0.987	± 0.15	0.7075	±0.01	0.5273	±0.05	0.1289	±0.019
**MRSA (Pus)**	1.062	± 0.08	0.6963	±0.01	0.527	±0.02	0.1328	±0.013
**MRSA (Catheter)**	0.921	± 0.07	0.6769	±0.09	0.5219	±0.02	0.1206	±0.018
**MSSA (Pus)**	1.1926	± 0.21	0.7383	±0.02	0.5216	±0.01	0.119	±0.009
**MSSA (Urine)**	1.0713	± 0.15	0.6547	±0.07	0.4899	±0.03	0.1164	±0.016

CCARM^a^ Culture Collection of Antimicrobial Resistant Microbes, Seoul, Korea

ATCC^b^ American Type Culture Collection

SD^c^ Standard Deviation

The SEM analysis of *S. aureus* biofilm treated with eugenol and control (untreated) biofilm are shown in Fig. 3C and D. In untreated samples, the biofilm was very organized in structure, with intact cell-to-cell connections (pointed by the arrow). The morphology of untreated *S. aureus* cells was smooth and regular, with an intact cell membrane. Conversely, the bacteria in eugenol-treated biofilms lost cell-to-cell connections. The organized structures of the biofilm were also disrupted in the eugenol-treated samples. From the rough and shrunken appearance of the cells, it is apparent that the eugenol-treated bacterial cells lost their normal morphology. The completely dead cells or cellular debris were identifiable by the pores in cell membrane (pointed by arrow). These results indicate that the biofilm-eradication potential of eugenol may be due to (1) disruption of the cell-to-cell connections and (2) cell lysis.

### Effect of eugenol on rate of kill of *S. aureus* in preformed biofilms

**Fig 4:**
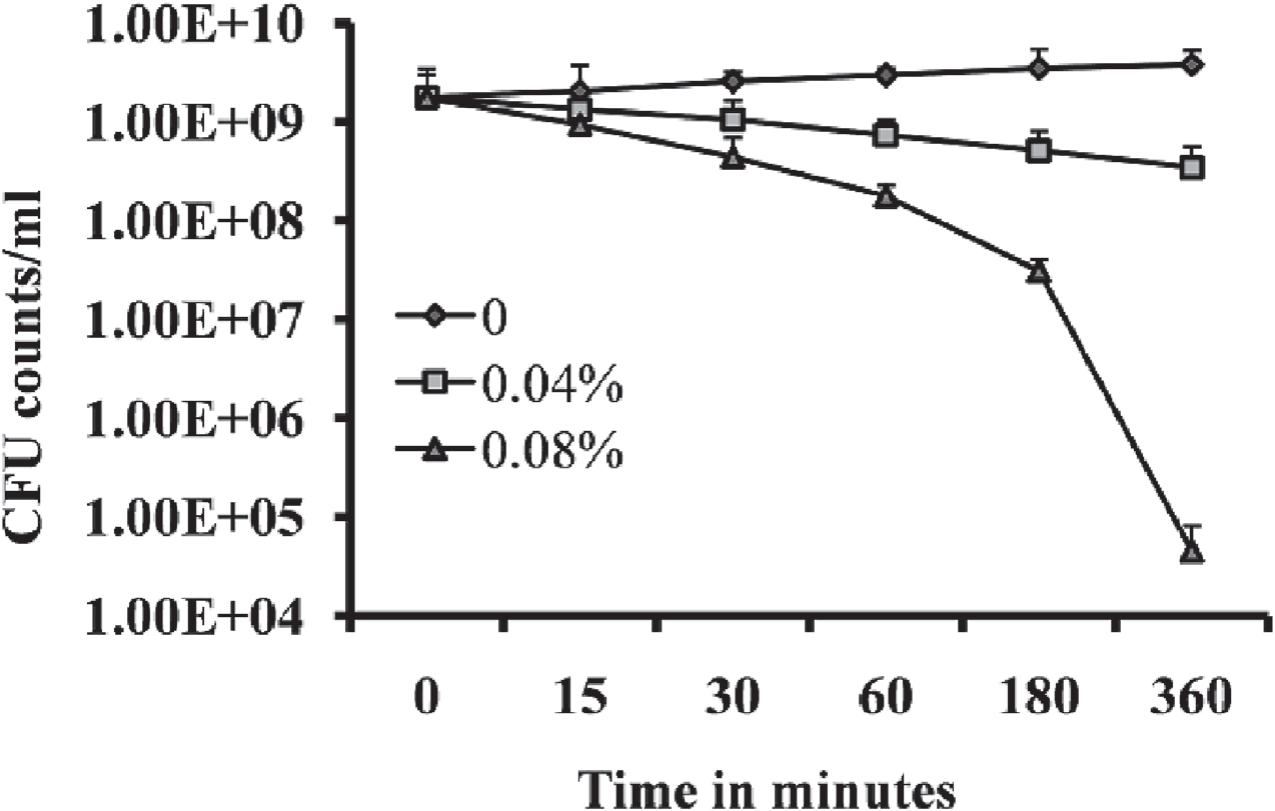
Time-course of the bactericidal effect within established *S. aureus* (ATCC 29213) biofilms. The pre-established biofilms were treated with MIC or 2×MIC eugenol and the cell viability was expressed as cfu counts at different time intervals. The control sample was untreated. The error bars represent standard deviation from mean value. The experiment was performed in triplicate and repeated 3 times.

Time-kill analysis was performed to determine the rapidity and duration of antibacterial activity of eugenol on *S. aureus* within the biofilms. Treatment of pre-established biofilms with 2 × MIC exhibited a strong bactericidal effect on *S. aureus* within biofilms. Within 3 and 6 h of treatment, counts of viable bacterial cells were decreased by more than two log 10 steps and four log 10 steps (Fig. 4). This observation suggests that the strong bactericidal activity of eugenol on *S. aureus* within biofilms reaches maximal effect within 3–6 h of incubation.

### Eugenol reduced *in vivo* colonization of *S. aureus*

**Fig 5:**
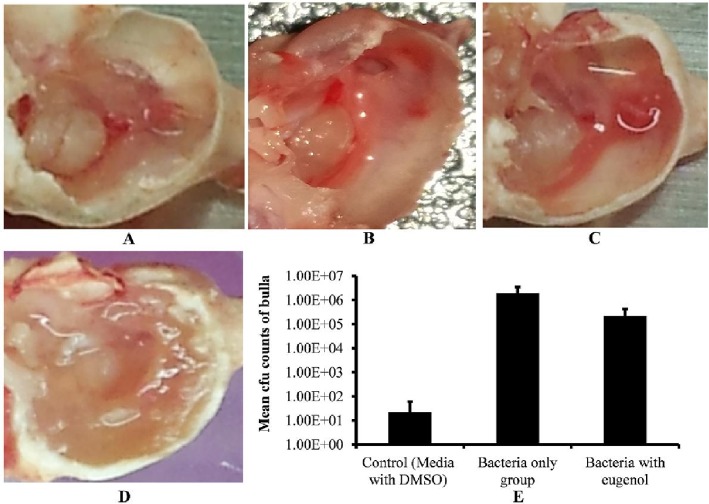
Effect of eugenol on colonization of *S. aureus* (ATCC 29213) *in vivo* in the middle ear of rat. (A) Image of no procedure control rat middle ear. (B) Image of rat middle ear inoculated with DMSO (0.1%). (C) Image of rat middle ear inoculated with bacteria and eugenol. (D) Image of rat middle ear inoculated with bacteria only. (E) Mean cfu counts of rat bullas inoculated with DMSO, bacteria only and bacteria with eugenol groups. The values are means of two independent experiments. The statistical significance was calculated using one-way analysis of variance (ANOVA) (P < 0.003).

After one week of inoculation, rats treated with bacteria alone, bacteria with eugenol (0.5× MIC), DMSO control or no procedure control were sacrificed and their bulla were collected and photographed (Fig. 5 A, B, C and D). The bulla of rats inoculated with bacteria only were completely filled with biofilm matrix, cell debris, and obstructed with swelling mucosa. Conversely, the bulla of rats inoculated with bacteria and eugenol were clear with no visible biofilm, matrix, or cell debris. Similarly the DMSO control and no procedure control bulla were also clean with no swelling mucosa.

The cfu counts of whole bulla lysates showed significantly lower number of *S. aureus* recovered from the bulla of rats inoculated with bacteria and eugenol in comparison to the counts from the group treated with bacteria alone. The mean cfu counts of group treated with bacteria only was 1.96 ×10^6^ (*SD* = 1,493,569), while cfu from group treated with bacteria and eugenol was 2.23 × 10^5^ (*SD* = 189,661.1). No bacteria were detected in most of the DMSO control bulla, however few bacteria 2.2 ×10^1^ (*SD* = 38), were observed in some bulla, which may be normal body flora. A significant (*p* < 0.003) 88% decrease in *S. aureus* colonization was observed in the middle ear of rat in the presence of eugenol (Fig. 5E).

**Fig 6:**
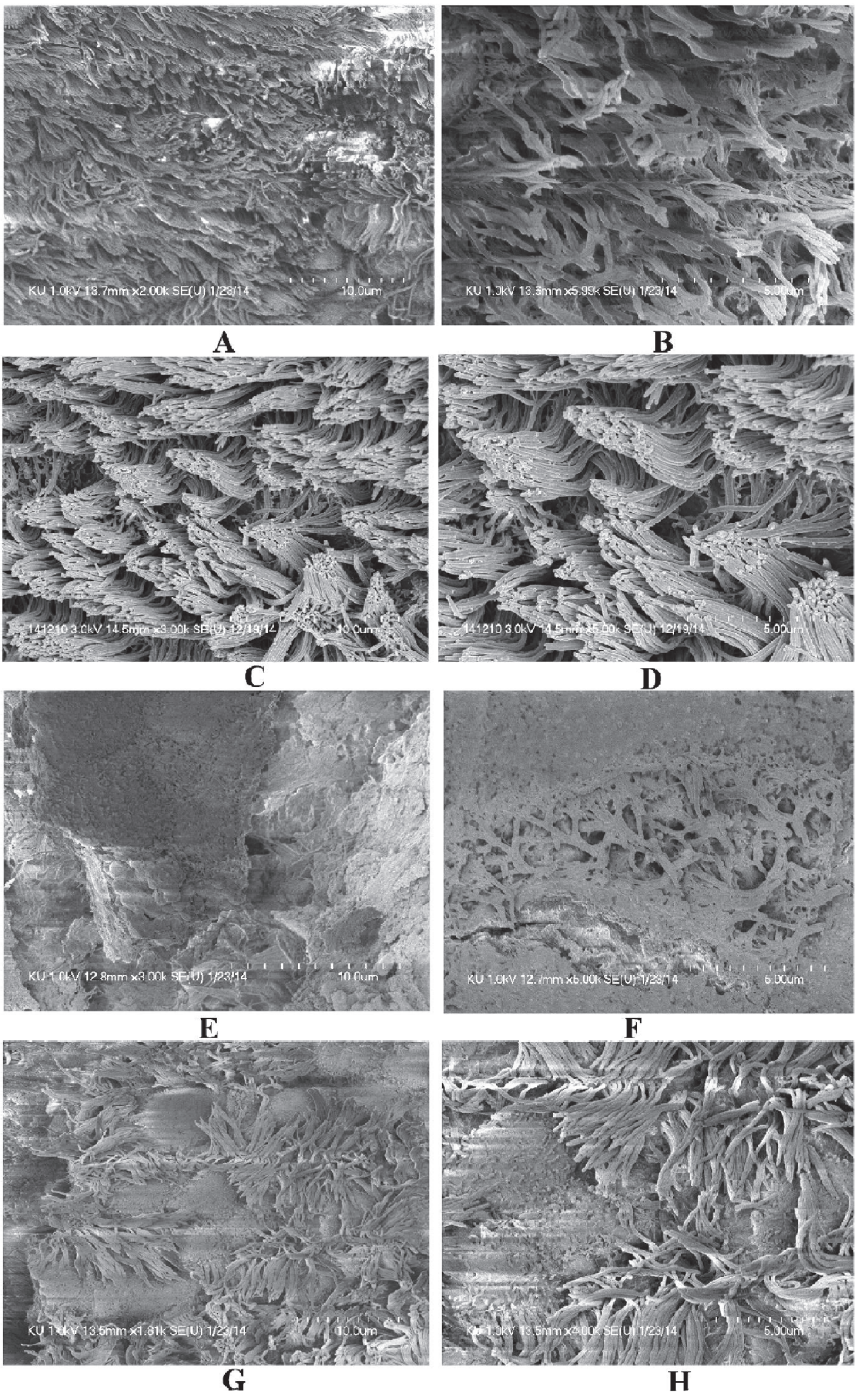
The effect of eugenol on colonization of *S. aureus* (ATCC 29213) in middle ear of rat *in vivo*. (A&B) Representative SEM images of no procedure control rat middle ear. (C & D) Representative SEM images of middle ear of rat inoculated with DMSO. (E & F) Representative SEM images of middle ear of rat inoculated with bacteria only. (G & H) Representative SEM images of middle ear of rat inoculated with bacteria and eugenol. Images scale bars correspond to 5 and 10μm.

The SEM images of middle ears of rats inoculated with bacteria only, bacteria with eugenol, DMSO control or no procedure controls are shown in Fig. 6. In the untreated (Figs. 6 A & B) and DMSO control middle ear (Fig. 6 C & D), the ciliated epithelium in the hypotympanum area and Eustachian tube orifice area are intact. In the group treated with bacteria only, the whole middle ear was covered with bacterial biofilms (Fig. 6 E & F). In these samples, cell debris and exopolysaccharide (EPS) of biofilms were deposited on the tip of the cilia, making the cilia invisible. Conversely, in bulla of rats treated with bacteria and eugenol, there was no visible biofilms formation or detectable cell debris in the middle ear. There was no deposition of debris or EPS on the cilia of the epithelium, although the cilia of the epithelium appeared conglomerated (Fig. 6 G & H).

### Eugenol decreases gene expression of biofilm and enterotoxin related genes

The quantification of *icaD*, *sarA* and *seA* gene expression in biofilm grown with eugenol in compare to control detected a significantly (*p* < 0.05) more 2-fold changes (Table 3). The intercellular adhesion (*icaD*) gene was significantly down regulated by 1.3, 6.1 and 3.4 folds in 24, 36 and 48 h biofilms. The expression of the staphylococcus enterotoxin A gene (*seA)* was significantly decreased by 1.3, 12.9 and 17.8 folds in 24, 36 and 48 h biofilms. Similarly, the *sarA* gene expression was also significantly decreased in biofilms grown over 24, 36 and 48 h by 1.5, 8.8, and 2.9-folds respectively. The down regulation of *icaD*, *sarA* and *seA* indicates that eugenol at sub-inhibitory concentration may decrease biofilm growth by reducing the expression of biofilm-related genes.

**Table 3:** Relative expression levels of *icaD*, *sea* and *sarA* in *S. aureus* (ATCC 29213) biofilms grown with eugenol at 0.5×MIC for 24, 36 and 48 h.

Genes	Gene function	Fold changes in 24 h	Fold changes in 36 h	Fold changes in 48 h
*icaD*	Intercellular adhesion (*icaD*)	−1.31 (*P* = 0.05)	−6.1 (*P* = 0.05)	−3.48 (*P* = 0.04)
*sarA*	Staphylococcal accessory regulator A	−1.49 (*P* = 0.02)	−8.8 (*P* = 0.04)	−2.79 (*P* = 0.05)
*seA*	Staphylococcus enterotoxin A	−1.33 (*P* = 0.04)	−12.9 (*P* = 0.02)	−17.85 (*P* = 0.02)

### Eugenol increases crystal violet absorption

**Fig 7:**
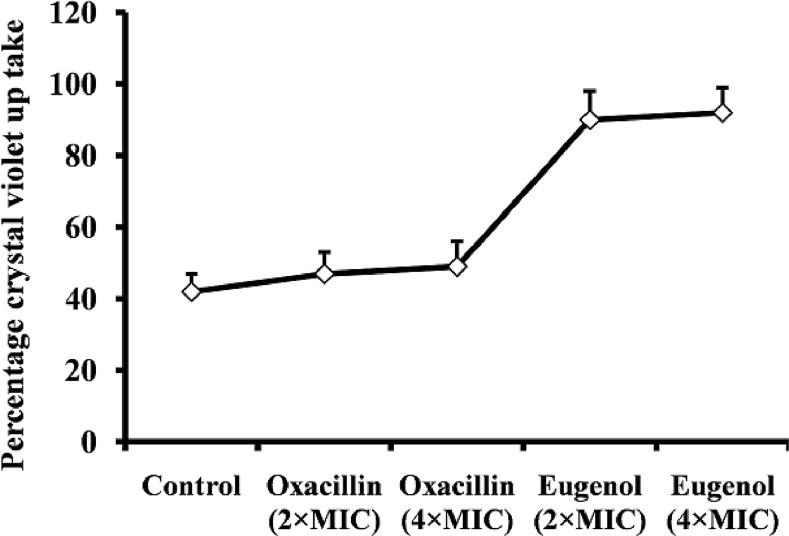
Crystal violet uptake of eugenol and oxacillin-treated *S. aureus* (ATCC 29213). The bacteria were treated with 2×and 4×MIC of eugenol or oxacillin and crystal violet absorption was assayed as show in the methods section. The mean ± SD for three replicates are presented.

The results of the crystal violet absorption assay indicate that eugenol alters membrane permeability of *S. aureus* and increases crystal violet absorption. The uptake of crystal violet by *S. aureus* was 42% in the absence of eugenol, but increased to 90% and 92% after MIC and 2×MIC eugenol treatments respectively (Fig. 7). Oxacillin used as a negative control elicited no detectable effect, indicating that it does not alter the membrane permeability.

### Eugenol acts synergistically with carvacrol to eradicate biofilm

**Fig 8:**
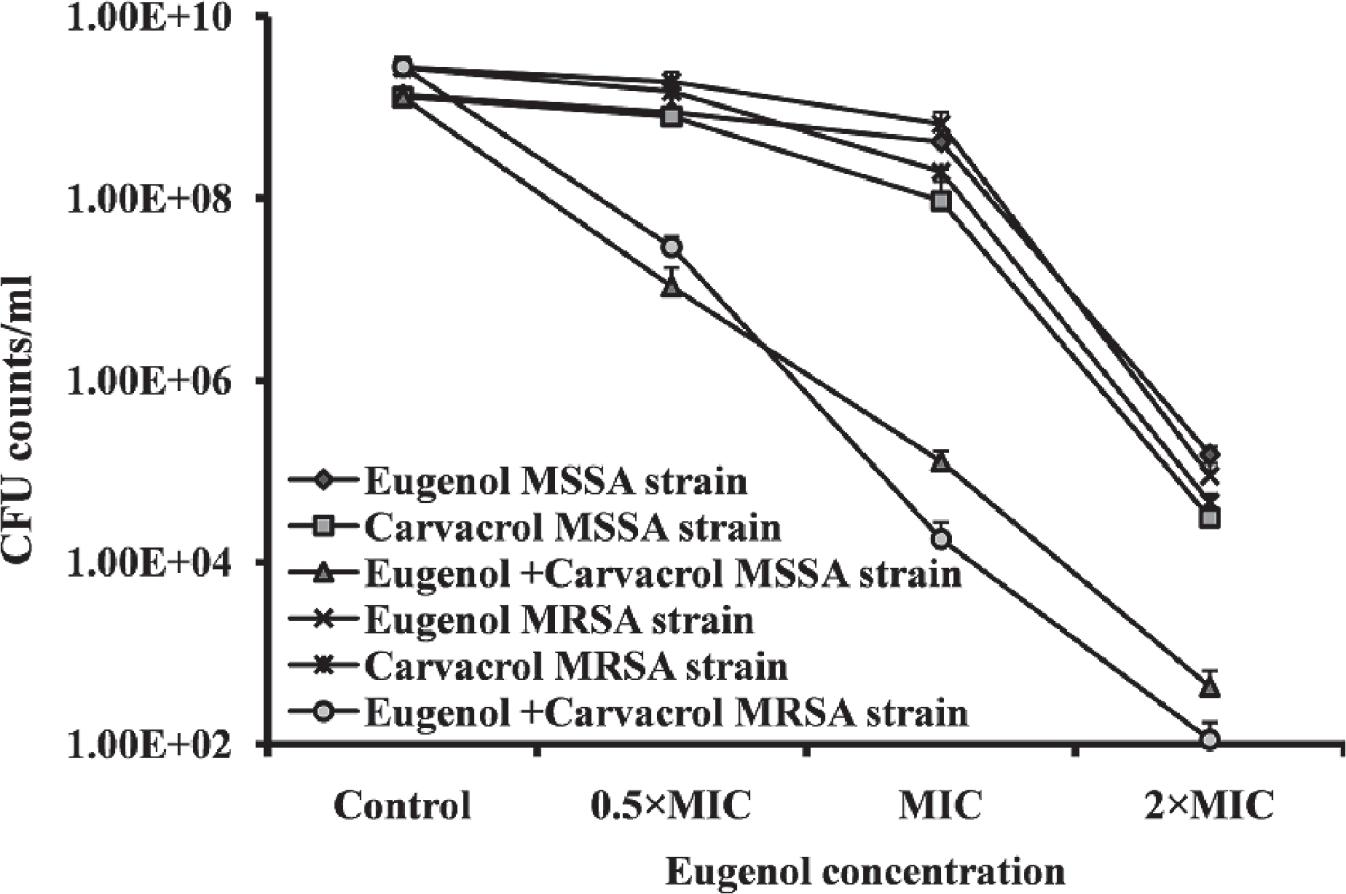
Synergistic effect of eugenol with carvacrol for eradication of preformed biofilm. The MRSA and MSSA pre-established biofilms were treated with different concentrations of eugenol or carvacrol alone or in combination, and the cell viability was determined by cfu counts. The experiment was performed in triplicates and repeated two times.

The combination of eugenol with carvacrol exhibited a synergistic effect against established MRSA and MSSA biofilms. MBEC of eugenol and carvacrol was determined to be 2×MIC (0.08% and 0.04%). Treatment of established biofilms with 0.25×MBEC (0.02%) of eugenol alone decreased the cfu counts by 25%. Similarly, 0.25×MBEC (0.01%) carvacrol decreased the viable bacteria by 40% in established biofilms. The combination of the two compounds (0.02% eugenol and 0.01% carvacrol) significantly decreased the bacteria in already established biofilms by 99%. The cfu counts of MRSA and MSSA biofilms were significantly decreased from 2.79×10^9^ and 1.32×10^9^ to 2.91×10^7^ and 1.07×10^7^ respectively (Fig. 8). When eugenol and carvacrol were used in combination, the MBEC was reduced ≥4-fold producing a synergistic effect as defined by FICI ≤0.5.

## Discussion

The paradigm of biofilm resistance presents a major hurdle for the treatment of a number of infectious disease types, increasing the economic burden, prolonging hospital stays, leading to recurrent infections, and increasing fatalities in the most recalcitrant cases [35]. The classical antibiotics target actively growing bacteria and are ineffective against the quiescent bacteria within biofilms [36]. Many phyto-compounds, including eugenol, have been reported to disrupt the bacterial membrane bilayer, cause membrane depolarization, and increase the membrane permeability in bacteria [28,37,38,39]. In the present study, we investigated the potential effects of eugenol on MRSA and MSSA clinical strains biofilms *in vitro* and bacterial colonization using the OM model *in vivo*.

**Fig 9:**
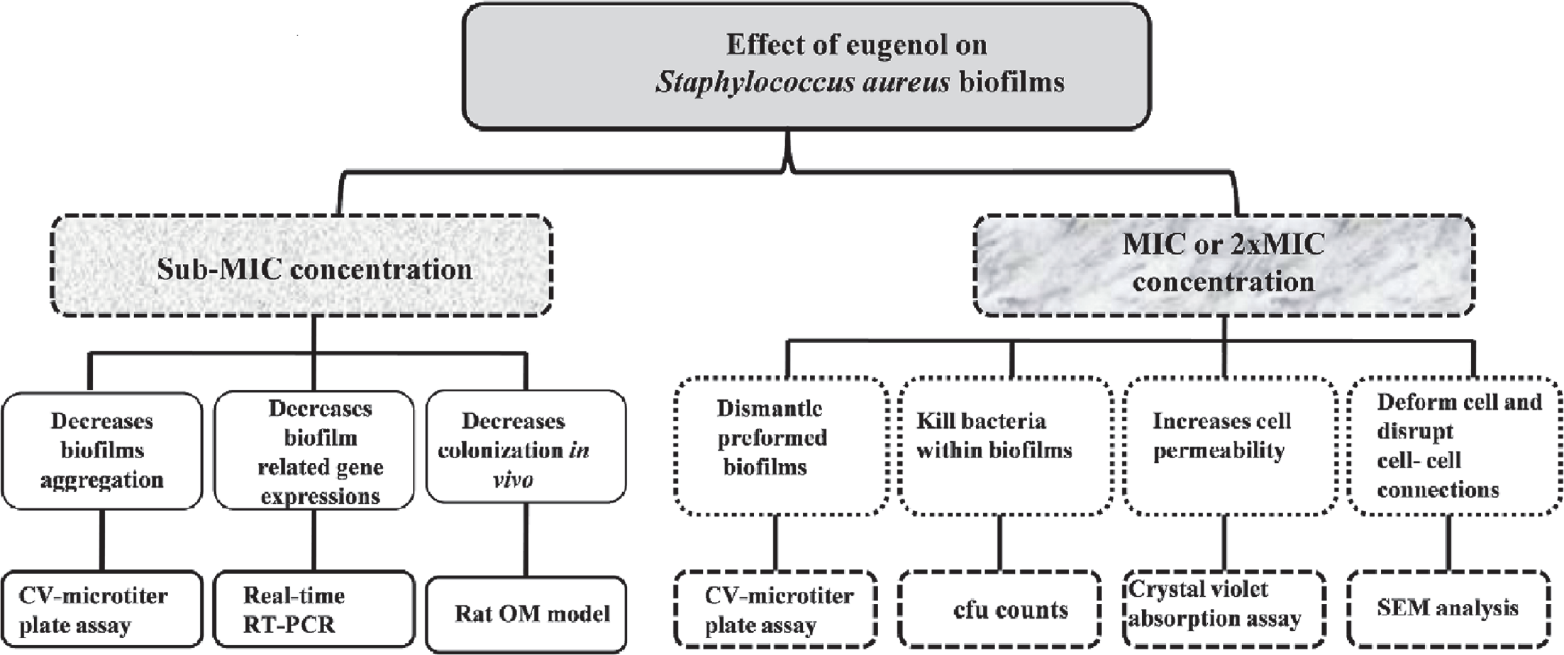
Systematic diagram showing effect of eugenol on *Staphylococcus aureus* biofilms.

Our results demonstrate significant inhibitory activity of eugenol on MRSA and MSSA biofilms *in vitro*. Eugenol can effectively eradicate pre-established biofilms, and decreases the expression of biofilm- and enterotoxin-related genes. It predominantly damages the cell membrane and causes the leakage of the cell contents. It significantly decreases the colonization of *S. aureus* in rat middle ear *in vivo* (Fig. 9). *Staphylococcus aureus* forms biofilms by two pathways; *ica*-dependent and *ica*-independent. The *ica*-dependent biofilm formation (in MSSA) is mediated by the polysaccharide intercellular adhesion or poly-N-acetylglucosamine (PIA/PNAG), which is synthesized by enzymes encoded by *ica*, *while the ica*-independent biofilm formation (in MRSA) is mediated by proteins binding to proteins of the extracellular matrix [10,11]. In this study eugenol is equally effective against MRSA and MSSA biofilms; therefore we have not considered this point. However, the biofilm experiments were carried-out in TSB medium supplied with 0.5% glucose, and it was previously reported that glucose can induce *ica*-independent biofilm formation in MRSA, probably mediated by proteins [10]. Therefore, it can be assumed that eugenol may be equally effective against *ica*-independent biofilm.

Eugenol significantly inhibits the growth of MRSA and MSSA biofilms in a concentration-dependent manner. At sub-MIC concentrations, >50% biofilm biomass was reduced in eugenol-treated samples, as compared to the control samples. The decreased cfu in eugenol-treated biofilms indicate that eugenol prevents cell aggregation and disrupts the organization of biofilms. These results were further supported by the SEM images, in which low numbers of adherent cells were visible. The inhibitory effect of eugenol on planktonic cell was low, as compared to biofilm inhibition. This is in line with other studies, reporting that phenol-containing phyto-compounds such as eugenol decrease biofilms growth *in vitro* [40].

Using the OM rat model, we analyzed the colonization of *S. aureus* in the middle ear of rats. The digital images of bulla of rats inoculated with bacteria and eugenol or DMSO only were clear in comparison to the bulla of rats treated with bacteria only. The bulla of rats treated with bacteria only were full of matrix, exhibited swollen mucosa, and significant presence of cell debris. The significantly decreased cfu counts and the tissue morphology in SEM images further support our results, showing that eugenol decreases *S. aureus* colonization in rat middle ear. Our results of *in-vivo* experiments demonstrate that eugenol possesses notable activity against colonization of *S. aureus* in middle ear. However, further study with suitable OM model is needed to elucidate the pre-established biofilm eradication activity of eugenol in the middle ear. Eugenol, the major phenolic compound and antimicrobial component of clove oil, has been registered in the European Union (EU) for use as a flavoring ingredient in foodstuffs and is considered to pose no risk to consumer health [41]. The eugenol structure or eugenol combined with other adjuvants can therefore be used against *S. aureus* biofilm infections. In particular, it may have potential for the treatments of OM where the liquid antibiotics are not permeable through the tympanic membrane. Previously it was reported that the vapors of essential oils can diffuse through the tympanic membrane and reach the middle ear to produce an antimicrobial effect on acute OM [42,43].

To elucidate the mechanism underlying the observed decreased biofilm growth in the presence of eugenol, we analyzed the gene expression of biofilm-related *sarA* and *icaD* genes. The staphylococcal accessory regulatory (*sarA*) locus encodes a DNA-binding protein (SarA) and has a global impact on gene expression and biofilm formation in *S. aureus [17,44,45]*. Similarly, the *icaD* is an important biofilm candidate gene. It regulates synthesis of an intercellular adhesion polysaccharide that supports cell-to-cell bacterial contacts by means of a multilayered biofilm [8,9]. In our current study, we detected a low expression of the *sarA* and *icaD* genes in biofilms grown with eugenol. This result indicates that eugenol interferes in the expression of these biofilm-related genes, resulting in decreased accumulation of polysaccharides and decreased adhesion of cells in biofilms *in vitro*. The low expression of *seA* gene in eugenol-treated biofilms indicates that eugenol is effective in decreasing the enterotoxins. Previously, Que et al. have reported down regulation of *sarA*, *ica*, and *seA* gene expression levels in the presence of eugenol in a stationary culture of *S. aureus* [29]. Similarly the decreased expression of *sarA* gene has been shown to contribute to low inflammation and colonization in biofilm formation *in vivo* [46].

Our results demonstrate significant biofilm-eradicating property of eugenol on MRSA and MSSA clinical strains. Eugenol treatment at concentration twice of MIC significantly decreased biomass and cell viability of established biofilms. This activity is maximal within 6 h of inoculation. The decrease in biomasses and cell viability indicates that eugenol eradicates *S. aureus* biofilm by two mechanisms; primarily it causes a lysis of bacteria within biofilms, and, secondary, it disrupts cell-to-cell connections and dismantles the biofilm organization. Eugenol is a phenolic compound, hydrophobic in nature, which primarily acts by disrupting the cytoplasmic membrane, resulting in the loss of normal shape of the bacteria, (resulting in shrunken appearance) and disruption of cell-cell connections [23]. These cell-to-cell connections are important for the formation of organized biofilm and bacterial colonization [47] and the disruption of these structures may result in the detachment of cells within biofilm, allowing them to be easily washed away. As a lipophilic compound, eugenol can pass through the cell wall and cytoplasmic membrane, disrupting the structure of different layers of polysaccharides, fatty acids, and phospholipids, permeabilizing the cell membrane, and resulting in cell lysis [48]. In the current study and others reports [38] depolarization and increased permeability were demonstrated in eugenol-treated bacteria. The severity of effect of eugenol on *S. aureus* biofilms results from the disruption of cell-to-cell connection, increased cell permeability causing leakage of internal cell contents to complete cell lysis, as seen in SEM analysis. Loss of membrane integrity and cell surface damage indicate that the bactericidal action of eugenol against *S. aureus* is elicited through the mechanism of membrane disruption and blocking of cell growth [28,41].

Carvacrol is a phenolic monoterpenoid and a major constituent of oregano, which has been reported to disintegrate the outer membrane of bacteria [49,50]. In current study, we detected a synergistic effect of eugenol with carvacrol against *S. aureus* biofilms. The MBEC was reduced ≥4-fold, suggesting a synergistic effect, as defined by the FICI≤0.5. Both eugenol and carvacrol have been shown to disrupt the cell membrane and depolarize the cell. Moreover, carvacrol at low concentration forms channels through the membrane by pushing apart the fatty acid chains of the phospholipids and thereby allowing ions to leave the cytoplasm [51]. Probably due to the structure differences, the two compounds interact in distinct ways with the cell membrane to enhance the biofilm eradication activity [52]. Further study is warranted to determine the exact mechanism underlying the synergistic effect of these two compounds on *S. aureus*.

## Conclusion

In conclusion, this study demonstrated that eugenol has excellent antibacterial activity, either alone or in combination with carvacrol, against MRSA and MSSA clinical strains biofilms. Eugenol was able to inhibit biofilm formation, disrupt the cell-to-cell connections, detach existing biofilms, and kill bacteria in biofilms of both MRSA and MSSA with equal effectiveness. It significantly decreased the expression of biofilm- and enterotoxin-related genes, and decreased bacterial colonization in the middle ear. Eugenol is safe to use and approved by EU and FDA as a food preservative. Therefore, eugenol administered alone or in combination with carvacrol could be used against *S. aureus* biofilm infections.

## Supporting Information

S1 Fig *In-vitro* biofilm formation capability of *S. aureus* clinical strains. (A) *In-vitro* biofilm formation ability of 72 clinical strains (43 MRSA and 29 MSSA) was detected by CV-microtiter plate assay. (B) 72 clinical strains divided into High, intermediate and low biofilm producer on basis of biofilm growth *in-vitro*
(TIF)

S2 Fig Effect of DMSO on *Staphylococcus aureus* and on Human middle ear epithelium cell line (HMEEC). (A) Optical density of *Staphylococcus aureus* ATCC 29213 strain grown with different concentration of DMSO for 24 h. (B) Absorbance at 450nm (detected by Cell Counting Kit-8) of HMEEC line grown with different concentrations of DMSO.
(TIF)

S3 Fig Hematoxylin and Eosin (H&E) histology of middle ear of rat inoculated with DMSO. Fig. A, showing images of the middle ear mucosa of no procedure control group. In no procedure control middle ear mucosa is composed of single layered epithelium and sub-epithelial tissue attached to bony bulla. Fig. B, C and D, showing images of the middle ears inoculated with DMSO. No difference in morphology or any widen space is visible between epithelium layer and bony bulla of DMSO treated group, indicating no cytotoxicity on middle ear epithelium.
(TIF)

S1 Table CCARM MRSA strains used in this study with antibiotic resistance profile. (DOCX)
