# Supplementary figures and images for: Eugenol: A Phyto-Compound Effective against Methicillin-Resistant and Methicillin-Sensitive Staphylococcus aureus Clinical Strain Biofilms
Source: PLoS One. 2015 Mar 17;10(3):e0119564. doi: 10.1371/journal.pone.0119564 (PMC4364371; doi:10.1371/journal.pone.0119564)

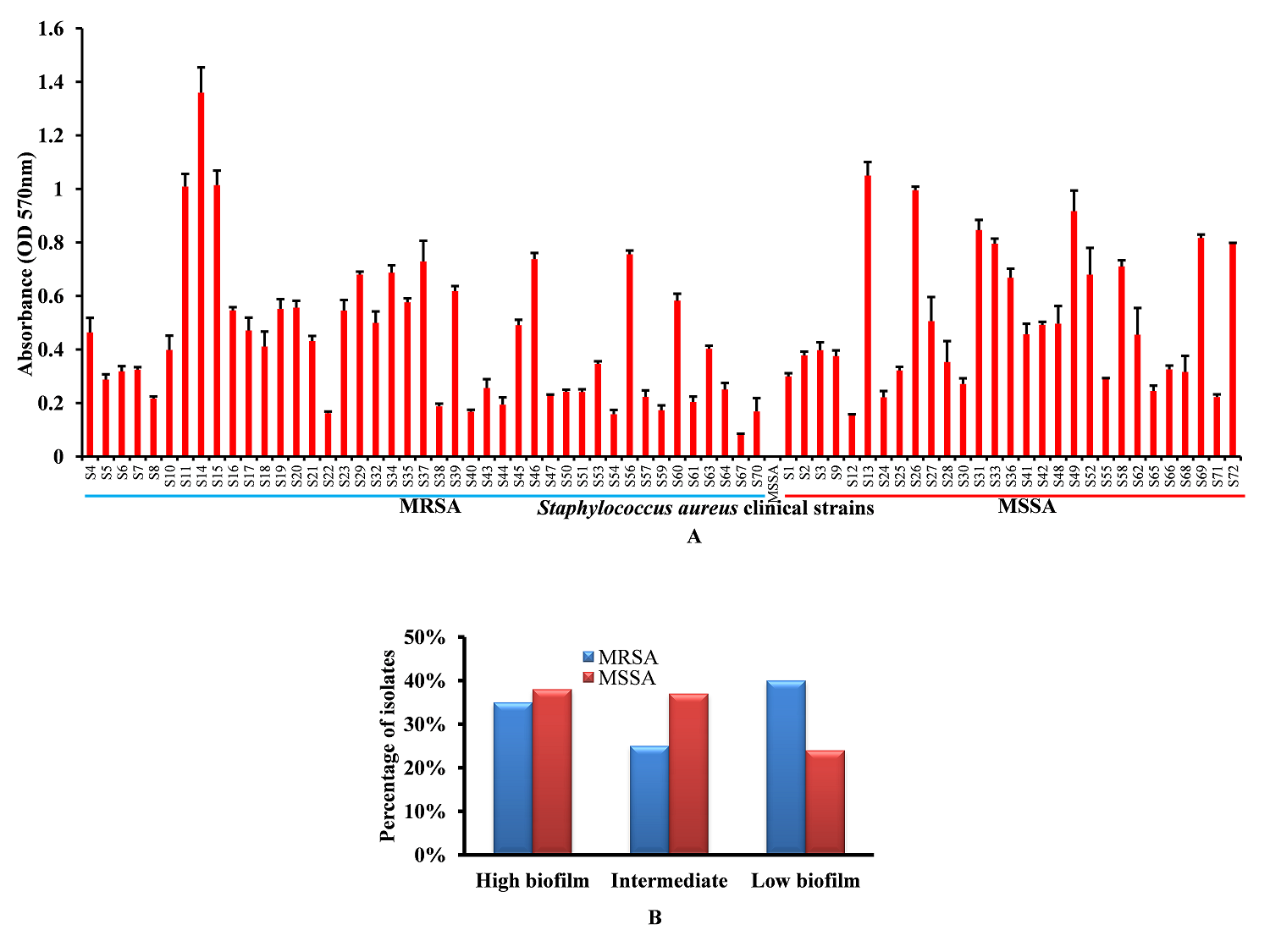

Supplement: S1 Fig — (A) In-vitro biofilm formation ability of 72 clinical strains (43 MRSA and 29 MSSA) was detected by CV-microtiter plate assay. (B) 72 clinical strains divided into High, intermediate and low biofilm producer on basis of biofilm growth in-vitro (TIF) [file pone.0119564.s001.TIF]

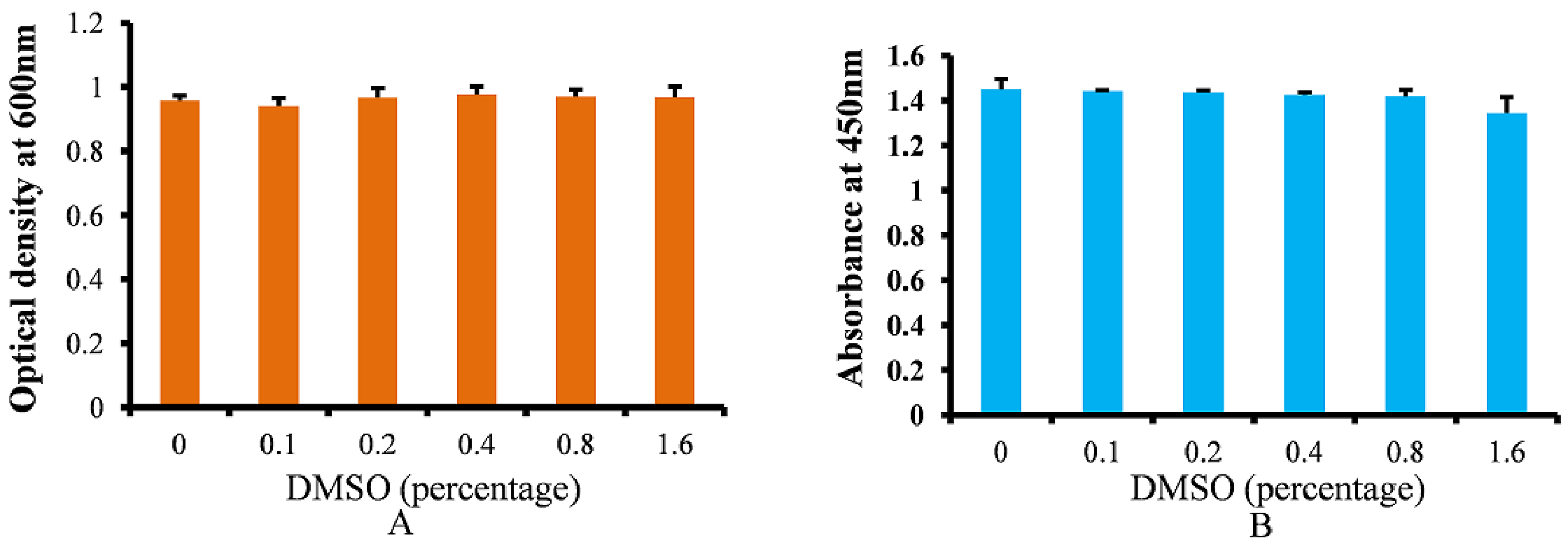

Supplement: S2 Fig — (A) Optical density of Staphylococcus aureus ATCC 29213 strain grown with different concentration of DMSO for 24 h. (B) Absorbance at 450nm (detected by Cell Counting Kit-8) of HMEEC line grown with different concentrations of DMSO. (TIF) [file pone.0119564.s002.tif]

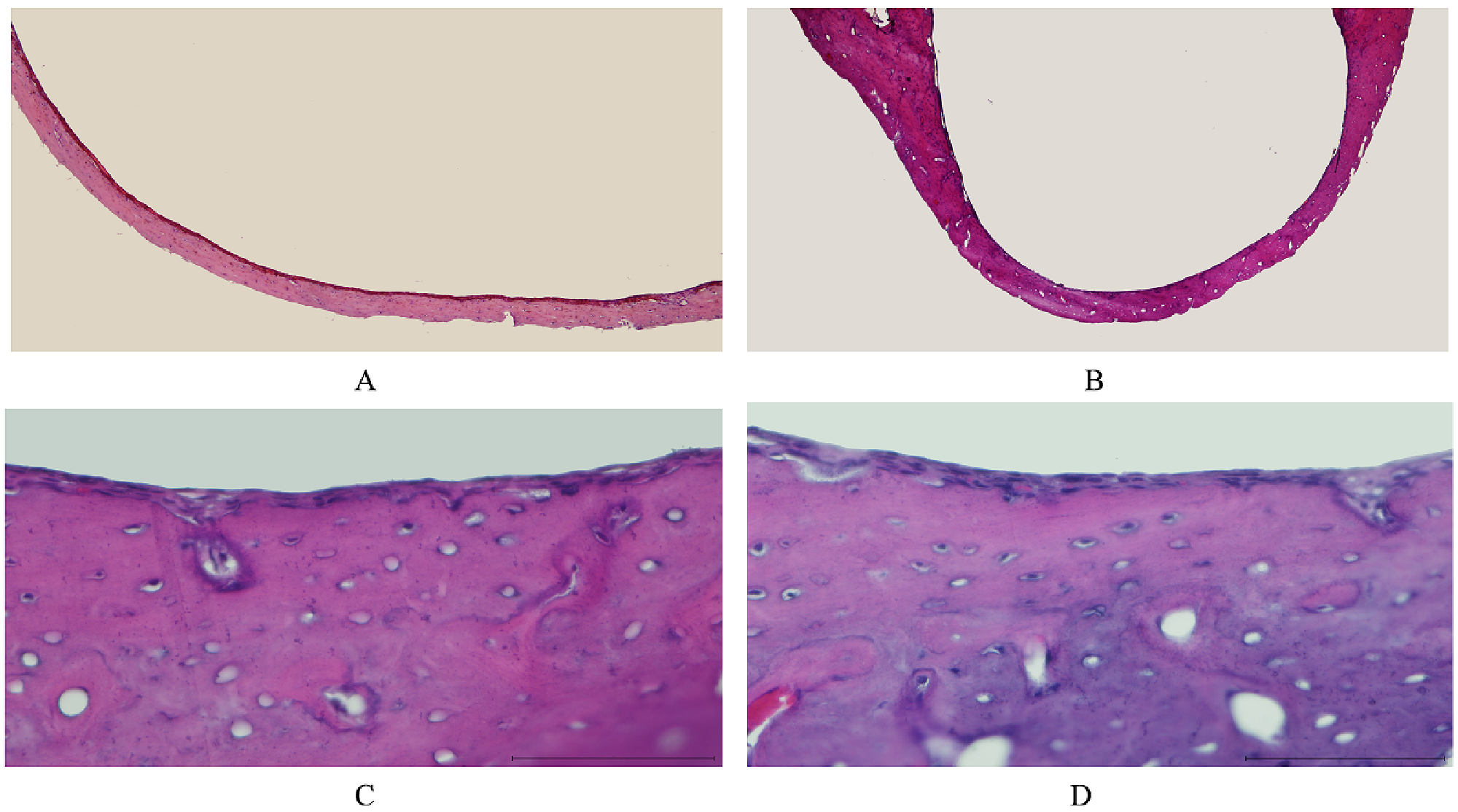

Supplement: S3 Fig — Fig. A, showing images of the middle ear mucosa of no procedure control group. In no procedure control middle ear mucosa is composed of single layered epithelium and sub-epithelial tissue attached to bony bulla. Fig. B, C and D, showing images of the middle ears inoculated with DMSO. No difference in morphology or any widen space is visible between epithelium layer and bony bulla of DMSO treated group, indicating no cytotoxicity on middle ear epithelium. (TIF) [file pone.0119564.s003.tif]
