# Supplementary material for: Eugenol: A Phyto-Compound Effective against Methicillin-Resistant and Methicillin-Sensitive Staphylococcus aureus Clinical Strain Biofilms
Source: PLoS One. 2015 Mar 17;10(3):e0119564. doi: 10.1371/journal.pone.0119564 (PMC4364371; doi:10.1371/journal.pone.0119564)
Supplement: S1 Table — (DOCX) [file pone.0119564.s004.docx]

|  |
| --- |

|  |
| --- |

**Table S1. CCARM MRSA strains used in this study with antibiotic resistance profile**

| **Antibiotics** | **CCARM 3903** | **CCARM 3108** | **CCARM 3969** | **CCARM 3912** | **CCARM 3807** |
| --- | --- | --- | --- | --- | --- |
| [CEP] Cephalothin | ---- | 128.0 µg/ml | ---- | --- | ---- |
| [CHL] Chloramphenicol | 2.0 µg/ml | ----- | 4.0 µg/ml | 2.0 µg/ml | 4.0 µg/ml |
| [CIP] Ciprofloxacin | 16.0 µg/ml | 8.0 µg/ml | ≥0.12 µg/ml | ≥0.12 µg/ml | 128.0 µg/ml |
| [CLI] Clindamycin | ≥128.0 µg/ml | ----- | ≥0.12 µg/ml | ≥128.0 | ≥128.0 µg/ml |
| [ERY] Erythromycin | ≥128.0 µg/ml | ≥128.0 µg/ml | ≥128.0 µg/ml | ≥128.0 µg/ml | ≥128.0 µg/ml |
| [GM] Gentamicin | ≥128.0 µg/ml | ≥128.0 µg/ml | 4.0 µg/ml | 16.0 µg/ml | 32.0 µg/ml |
| [NOR] Norfloxacin | 64.0 µg/ml | 32.0 µg/ml | 0.5 µg/ml | 0.5 µg/ml | ≥128.0 µg/ml |
| [OXA] Oxacillin | ≥128.0 µg/ml | ≥128.0 µg/ml | 4.0 µg/ml | ≥128.0 µg/ml | ≥128.0 µg/ml |
| [PEN] Penicillin | 32.0 µg/ml | ---- | 8.0 µg/ml | 8.0 µg/ml | 16.0 µg/ml |
| [RIF] Rifampin | ≤0.12 µg/ml | ---- | ≥0.12 µg/ml | ≥0.12 µg/ml | ≥0.12 µg/ml |
| [SXT]Trimethoprim-sulfamethoxazole | ≥128.0 µg/ml | ----- | 0.5 µg/ml | 0.5 µg/ml | 0.5 µg/ml |
| [TET] Tetracycline | 128.0 µg/ml | ----- | ≥0.12 µg/ml | ≥0.12 µg/ml | 16.0 µg/ml |
| [TEI] Teicoplanin | ---- | 2.0 µg/ml | ---- | ---- | ---- |
| [VAN] Vancomycin | 1.0 µg/ml | 2.0 µg/ml | 0.5 µg/ml | 1.0 µg/ml | ≥0.12 µg/ml |
